# Shade tree diversity and aboveground carbon stocks in *Theobroma cacao* agroforestry systems: implications for REDD+ implementation in a West African cacao landscape

**DOI:** 10.1186/s13021-016-0061-x

**Published:** 2016-08-24

**Authors:** Evans Dawoe, Winston Asante, Emmanuel Acheampong, Paul Bosu

**Affiliations:** 1 Faculty of Renewable Natural Resources (FRNR), Kwame Nkrumah University of Science and Technology (KNUST), Kumasi, Ghana; 2 SNV Netherlands Development Organization, Accra, Ghana; 3 Forestry Research Institute of Ghana (FORIG), Council for Scientific and Industrial Research (CSIR), Kumasi, Ghana

**Keywords:** Shade trees, *Theobroma cacao*, Species richness, Diversity indices, Carbon stocks, REDD+

## Abstract

**Background:**

The promotion of cacao agroforestry is one of the ways of diversifying farmer income and creating incentives through their inclusion in REDD+ interventions. We estimated the aboveground carbon stocks in cacao and shade trees, determined the floristic diversity of shade trees and explored the possibility of implementing REDD+ interventions in cacao landscapes. Using replicated multi-site transect approach, data were collected from nine 1-ha plots established on 5 km long transects in ten cacao growing districts in Ghana West Africa. Biomass of cacao and shade trees was determined using allometric equations.

**Results:**

One thousand four hundred and one (1401) shade trees comprising 109 species from 33 families were recorded. Total number of species ranged from 34 to 49. *Newbouldia laevis* (Bignoniacea) was the most frequently occurring specie and constituted 43.2 % of all shade trees. The most predominant families were Sterculiaceae and Moraceae (10 species each), followed by Meliaceae and Mimosaceae (8 species each) and Caesalpiniacaea (6 species). Shannon diversity indices (H’, H_max_ and J’) and species richness were low compared to other similar studies. Shade tree densities ranged from 16.2 ± 3.0 to 22.8 ± 1.7 stems ha^−1^ and differed significantly between sites. Carbon stocks of shade trees differed between sites but were similar in cacao trees. The average C stock in cacao trees was 7.45 ± 0.41 Mg C ha^−1^ compared with 8.32 ± 1.15 Mg C ha^−1^ in the shade trees.

**Conclusions:**

Cacao landscapes in Ghana have the potential of contributing to forest carbon stocks enhancement by increasing the stocking density of shade trees to recommended levels.

**Electronic supplementary material:**

The online version of this article (doi:10.1186/s13021-016-0061-x) contains supplementary material, which is available to authorized users.

## Background

The cacao sector in Ghana plays a significant role in the fight against poverty and the development of the economy of Ghana as a whole. Globally, Ghana is the world’s third largest producer (after Cote d’Ivoire and Indonesia) and has maintained its position as the second largest exporter of cacao beans after Cote d’Ivoire for the period 2005–2011 [1]. Cacao is Ghana’s leading cash crop and the highest export crop earner accounting for 8.2 % of the country’s gross domestic product (GDP) and 30 percent of total export earnings in 2010 [2]. In terms of employment, the livelihood of about six million people (25–30 % of the population) depends on the cacao sector [3].

Ghana’s cacao production is characterized by small-scale farming with an average productive cacao area per household of approximately 2–3 ha [1, 4]. Approximately 800,000 families grow cacao on 1.6 million hectares of land with the Western region having the highest production value (over 50 % of total production), followed by the Ashanti region (accounting for about 16 % of total production). The Eastern and Brong-Ahafo regions together account for about 19 % of total production [1].

There is a general belief that cultivation of cacao, as traditionally practiced in Africa, has the potential to restore carbon stocks to levels comparable to that in the native forest which they replaced. Cacao is an understory species and so cultivation is traditionally done under tree canopy. It is therefore intuitive that carbon stocks in cacao farms with dense tree canopy cover will be much similar to that in the original native forest than in farms with lower or no canopy shade. Indeed, several studies have revealed that a larger proportion of the carbon stocks in most cacao agroforests are contributed by the shade trees [5–8]. The question is whether carbon stocks in these emerging cacao plantations will be within reasonable proportion of the original native forests they have replaced. Understanding carbon accumulation dynamics in cacao agro-ecosystems is important to the long-term management of cacao farms that inure to climate change mitigation and the associated socioeconomic effects.

Although multi-strata shaded-cacao systems still occur in Ghana, there has been an increasing move towards intensification of cacao management with shade tree removal and monoculture practices [9, 10]. In the Western Region of Ghana, the majority of cacao farms are now predominantly being managed with low shade or no shade [11, 12]. The need to introduce approaches that combine supporting local cacao livelihoods for poverty alleviation and reducing ecosystem degradation in cacao production systems in Ghana is imperative. This is expected to be achieved through actions that improve business development skills of farmers, increase cacao productivity, enhance natural resources management, conserve biodiversity, and reduce emissions associated with smallholder cacao farming systems. It is expected that through this initiative, smallholder cacao farmers in Ghana can qualify and earn additional income through the REDD+ (Reduced Emissions from Deforestation and Forest Degradation) mechanism and related incentive packages associated with carbon mitigation programmes. Thus, information relating to carbon stocks especially from shade trees, is vital to the design of any mitigation intervention. However, though some research efforts have focused on carbon stocks and tree diversity within cacao farms, most of these data sets show results of selected farms [13, 14], with no clear indication of landscape level play out. There is also no information on the potential role of the cacao systems at the landscape level in future climate change mitigation mechanisms based on the prevailing site level conditions in the cacao systems, though these future mitigation mechanisms are being conceived at the landscape level for implementation.

The overall objective of this study was to quantify the baseline carbon stocks, tree diversity and shade tree crown cover variation associated with smallholder cacao agroforestry systems in the cacao landscape of Ghana. Specifically, we estimated the baseline aboveground carbon stocks in shade and cacao trees, explored the relationships between tree parameters and carbon storage and determined floristic diversity of shade trees. We hypothesized that cacao landscapes in Ghana have the potential of contributing positively to the country’s REDD+ efforts.

## Methods

### Study area

**Fig. 1:**
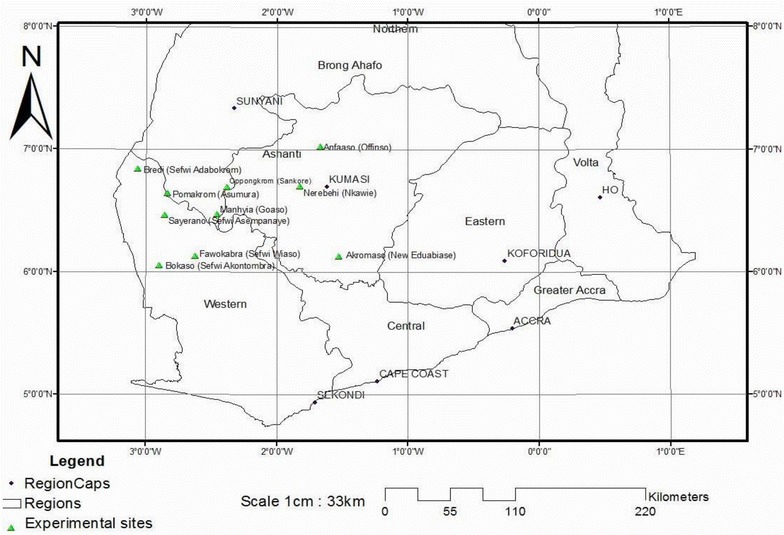
Locations of study communities and their administrative regions

The study was conducted in the mid-western cacao growing areas of Ghana covering three of the five cacao-growing regions namely, Western, Ashanti, Brong-Ahafo and regions, located between longitude 01° 30′ and 30 5′ W and latitude 06° 0′ and 07° 0′ N (Fig. 1). The area lies within the dry and moist semi-deciduous forest zones, characterized by the wet semi-equatorial climatic conditions with a double maxima rainfall (April–July and September–November) ranging between 1700 and 1850 mm per annum. The main dry season lasts from December to mid-March Average ambient temperatures are uniformly high throughout the year and average 26.0 °C. The predominant vegetation in the study districts is the semi-deciduous forest type, which is characterized by predominantly *Celtis*-*Triplochiton* association as described by [15], with some of the trees in the upper and middle layers shedding their leaves in the dry season. The cacao agroforestry systems in the study districts (political/administrative area where the sites are located) are mostly mixed stands of cacao and with variable proportions of naturally generated upper canopy shade trees such as *Terminalia superba* Engl. & Diels, *Triplochiton scleroxylon* K. Schum., *Alstonia boonei* de Wild and *Ceiba pentandra* (L.) Gaertn. Fruit trees such as orange (*Citrus sinensis* L.) Osbeck, Avocado (*Persea americana*) and mango (*Mangifera indica* L.), for shade, food and other purposes may be planted. The soils of the study area are from weathered phyllites and dominated by ochrosols (to the north) and ochrosol-oxisol intergrade (Rhodic Ferralsol) to the south [16]. They are generally deep, moderately well drained and brashy with a silty-loam humus texture in the 0–15 cm soil layer, which gives it a high moisture retention capacity [17].

Land tenure arrangements in the study area can be classified into farmers who own their land, family lands, rented lands and lands under sharecropping arrangements. Nearly half of all cacao farmers in the area own the cacao farmlands themselves with about 22 % being family lands and 33 % managed under sharecropping arrangements [18].

### Study site selection

Selection of study sites (the location/situate of transects) was based on the biophysical and socioeconomic diversity of the landscape. Ten (10) cacao growing districts were selected, three each in the Ashanti and Brong-Ahafo Regions, and four in the Western Region after an initial reconnaissance of the study area. In each district, one of several communities (nearest town/village within whose immediate environs the transects were laid) with contiguous cacao plantations was randomly selected as a focal or reference community (Fig. 1).

### Plot installation and data collection

**Fig. 2:**
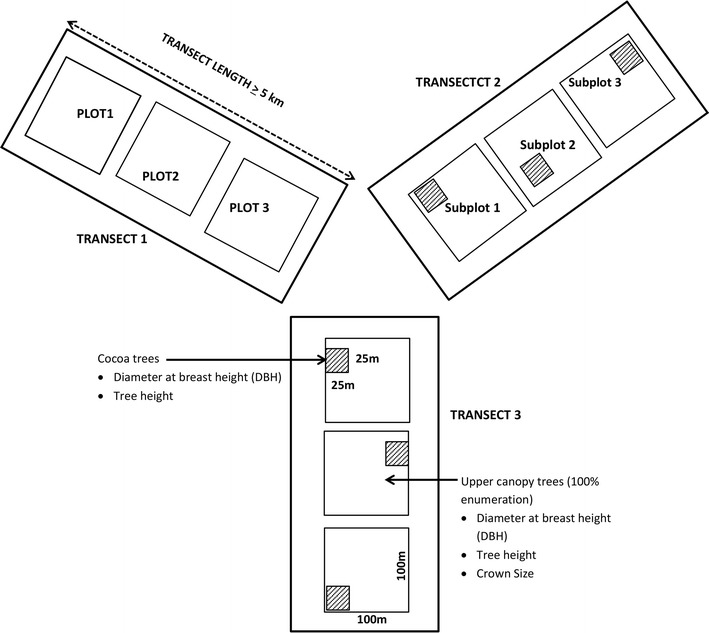
Layout of transects, main plots (100 m × 100 m) and subplots (25 m × 25 m) in each study district. *Lower arm* of *Y*-shape (Transect 3) shows dimensions of main and subplots and parameters measured (for both cocoa and shade trees) within each transect. Each transect is 5 km long and minimum distance between plots is 500 m

In each study community, three transects (laid out in a Y-shaped fashion and roughly aligned at 120° from each other) were established in a contiguous cacao landscape (Fig. 2). Each transect was at least 5 km long. Three plots, each measuring 100 m × 100 m, were established along each transect at intervals of at least 500 m. There were nine plots per site giving a total of ninety plots across all sites. In cases where the recording plot fell within a shade land use, the transect continued till the next cacao establishment. Within each 100 m × 100 m plot, subplots measuring 25 m × 25 m were demarcated. The subplots were placed at alternate points along the main transect line (Fig. 2). Plot level assessments included measurements of cacao (on 25 m × 25 m subplots) and shade canopy trees (100 m × 100 m main plots). All shade trees with diameter at breast height (DBH = 1.3 m) of 5 cm and above were identified (scientific and local names) by an experienced botanist. Subsequently tree botanical composition, population and spatial structure in the study districts were compared. Tree DBH was measured by calipers while heights were measured using a laser hypsometer (Nikon Laser Hypsometer). To obtain an estimate of the shade provided by the shade trees to cacao and their contribution to canopy cover, crown area of each shade tree within the 1 ha sample plot of cacao farms was estimated by measuring the diameter of the crown in eight different directions, following the cardinal points and a subdivision within the cardinal points, i.e. North-South, East-West and then North-West, North-East, South-West and South-East. The total crown cover for all the upper canopy trees was expressed as a percentage of 1 ha to ensure easy comparison with the parameters of the national forest definition at the plot level. All data collection took place in April and May 2014.

### Tree diversity assessment

The Shannon-Weiner diversity indices (H’, H max and J) were calculated using Biodiversity Professional Software (Version 2.) as follows:

1 $$H^{\prime} = - \mathop \sum \limits_{i = 1}^{S} (p_{i} \times \ln p_{i} ) = - \mathop \sum \limits_{i = 1}^{S} \left(\frac{{n_{i} }}{N} \times \ln \frac{{n_{i} }}{N}\right)$$

where *S* is the total number of species in the landscape, *N* is the total number of individuals, and *n*_*i*_ is the number of individuals of the ith species, $\frac{{n_{i} }}{N}$ is equivalent to *p*_*i*_, the probability of finding the i-th species. H max the maximum possible value of H’, and J’ the evenness (H’/H max) were also calculated. Spearman’s rank correlation test of species similarity between the ten sites was also carried out using biodiversity professional software.

### Above ground biomass and C-stocks estimation

We estimated aboveground biomass (AGB) of cacao and upper canopy trees using the allometric equation developed by [19] for moist forests:

2 $${\text{Biomass }}\left( {\text{kg}} \right) \, = { \exp } - 2. 9 7 7 { } + { \ln }\left( {{\text{X}}*{\text{Z}}^{ 2} *{\text{W}}} \right),$$

where X = species specific density; Z = diameter at breast height (DBH); and W = height. Species-specific wood densities were obtained from the World Agroforestry Centre’s Wood Density Database [20]. If a species was not listed, we computed the average wood density of the plot and assigned it to the unknown species. To calculate carbon stocks in shade or cacao trees, we applied the general conversion factor of 0.5 to ABG tree biomass [21].

### Statistical analysis

Percentage crown cover and carbon stocks were compared using the pooled data at the transect level as replicates for each site. For each variable (crown cover, cacao carbon, shade tree carbon and stem diameter) normal distribution was tested using the Shapiro-Wilks W-test for homogeneity of variances. Variables that conformed to normal distribution were analyzed using one-way analysis of variance (ANOVA) while those that did not meet the assumptions for an ANOVA even when transformed were analyzed using Kruskal–Wallis parametric ANOVA on the mean ranks using the software package Statistix 8.0 for windows [22]. Separation of means was done at 5 % probability level using Tukey’s HSD test. Regression and correlations analyses were also employed as tools for statistical tests and to establish trends and relationships between crown cover and carbon stocks, and shade tree diameter and carbon stocks and Shannon-Weiner Diversity Indices H’ H max and J.

## Results

### Abundance and composition of shade species and families in the cacao landscape

A total of 1401 individual trees (shade) were recorded across all the 10 study sites (Table 1). These comprised 109 different species from 33 families on 90 ha (average 15 trees per hectare) surveyed. Out of the 109 species, the total number of species encountered in the districts ranged from 34 at Sewfi Wiawso to 49 at Goaso. *Newbouldia laevis* (family *Bignoniacea*) was the most frequently occurring species and constituted 43.2 % of all shade trees in the districts but was most dominant in four districts. The most predominant families across the landscape were *Sterculiaceae* and *Moraceae* (10 species each), followed by *Meliaceae* and *Mimosaceae* (eight species each) and *Caesalpiniacaea* (six species). *Anacardiaceae, Euphorbiaceae* and *Palpilioniaceae* were each represented by five species. Ten families were represented by two or three species. Table 2 shows the most dominant shade tree species in each district.

**Table 1:** Overview of shade tree species diversity and selected dendrometric parameters in the different study districts

	Tree abundance^a^	Species richness^b^	Shade tree stem density (ha^−1^)	Mean tree height (m)	Mean stem diameter (cm)	Shannon H’	Shannon H max	Shannon J’
Adabokrom	97	35	10.8 ± 1.27	15.1 ± 0.74	51.6 ± 3.67	1.42	1.54	0.92
Akontombra	84	27	9.33 ± 1.22	11.9 ± 1.05	31.2 ± 4.71	1.22	1.43	0.85
Asempaneye	128	45	14.2 ± 1.11	19.1 ± 1.41	47.7 ± 3.91	1.51	1.65	0.92
Asumura	147	37	16.3 ± 2.58	13.8 ± 1.19	35.0 ± 3.97	1.04	1.57	0.66
Goaso	146	49	16.2 ± 3.00	15.3 ± 1.24	43.0 ± 3.55	1.52	1.69	0.90
New Edubiase	184	43	20.6 ± 3.25	18.2 ± 0.99	44.9 ± 2.85	1.34	1.63	0.82
Nkawie	136	40	15.1 ± 2.47	12.8 ± 0.64	36.6 ± 3.31	1.40	1.60	0.88
Offinso	204	46	22.8 ± 1.71	13.5 ± 0.82	40.2 ± 3.68	1.29	1.66	0.77
Sankore	108	35	12.0 ± 2.71	18.2 ± 1.58	49.6 ± 3.64	1.39	1.54	0.90
Wiawso	165	34	18.3 ± 3.57	13.7 ± 1.42	33.1 ± 3.72	0.99	1.53	0.65

^a^Total number of stems enumerated on a total plot size of 9 ha. These comprise all timber and non-timber tree species

^b^Number of different individual tree species recorded on a total plot size of 9 ha

**Table 2:** Dominant shade tree species recorded in each study district (percentages are proportions of total individuals recorded in each district)

Community	Most dominant species	Tree abundance^a^	Proportion of total individual shade trees (%)
Adabokrom	*Ficus exasperata*	97	10
Akontombra	*Persea americana*	84	16.7
Asempaneye	*Triplochiton scleroxylon*	128	10.2
Asumura	*Newbouldia laevis*	147	47.6
Goaso	*Milicia excelsa*	146	9.5
New Edubiase	*Newbouldia laevis*	185	24.3
Nkawie	*Morinda lucida*	136	13.2
Offinso	*Citrus sinensis*	205	24.0
Sankore	*Newbouldia laevis*	108	11.1
Wiawso	*Newbouldia laevis*	165	47.3

^a^Total number of stems enumerated on a total plot size of 9 ha

The average stand density of the shade trees varied and ranged from 9.33 ± 1.22 stems ha^−1^ for Akontombra to 22.8 ± 1.71 stems ha^−1^ for Offinso with an average of 15.6 ± 1.34 stems ha^−1^ across districts. The total number of species was highest in Goaso, where the Shannon-Weiner indices (Shannon H’ = 1.52, Shannon H max = 1.69 and Shannon J’ = 0.90) were higher than all the other locations. Wiawso had the lowest indices (Shannon H’ = 0.99, and Shannon J’ = 0.65). The Spearman’s rank correlation test of species similarity between the ten sites are shown in Table 3. The highest similarity index was 0.7 and the lowest 0.4.

**Table 3:** The Spearman’s rank correlation test of species similarity between study districts in Ghana

Communities	Adabokrom	Akontombra	Asempaneye	Asumura	Goaso	New Edubiase	Nkawie	Offinso	Sankore	Wiawso
Adabokrom	1	*	*	*	*	*				
Akontombra	0.6	1	*	*	*	*	*	*	*	*
Asempaneye	0.6	0.4	1	*	*	*	*	*	*	*
Asumura	0.6	0.6	0.5	1	*	*	*	*	*	*
Goaso	0.7	0.5	0.5	0.7	1	*	*	*	*	*
New Edubiase	0.7	0.6	0.5	0.6	0.7	1	*	*	*	*
Nkawie	0.6	0.5	0.5	0.6	0.6	0.6	1	*	*	*
Offinso	0.5	0.5	0.4	0.5	0.5	0.5	0.5	1	*	*
Sankore	0.6	0.6	0.5	0.6	0.6	0.6	0.5	0.6	1	*
Wiawso	0.5	0.7	0.4	0.5	0.5	0.5	0.5	0.5	0.5	1

### Relative abundance of timber and non-timber 1 species in the cacao landscape

All shade trees identified in the plots were grouped into timber and non-timber species. Generally, the relative abundance of non-timber species in all the districts was higher than timber species, except Asempaneye where the abundance of timber and non-timber species were equal (Table 4). In addition to being important as shade trees for cacao, the most important uses of shade trees in cacao systems were for their productive values e.g. fruit, timber, firewood, fodder and medicines (Additional file 1: Appendix 1). Apart from the fruit trees, (*Persea americana*, *citrus* spp. and the oil palm *Elieas guineensis*) which were planted by farmers, most of the shade trees were selectively left on farmlands during land preparation and nurtured by farmers during cacao establishment. A list of current and potential uses of all identified non-timber trees is given in Additional file 1: Appendix 1.

**Table 4:** Relative abundance of timber and non-timber species within smallholder cacao systems in ten cocoa growing districts in the high forest zone, Ghana

District	Timber species^a^	Relative abundance (%)	Non timber species^a^	Relative abundance (%)
Adabokrom	45	45.0	55	55.0
Akontombra	19	22.6	65	77.4
Asempanaye	64	50.0	64	50.0
Asumura	39	26.6	108	73.5
Goaso	67	45.9	79	54.1
New Edubiase	85	45.9	100	54.1
Nkawie	48	35.3	88	64.7
Offinso	44	21.6	160	78.4
Sankore	31	28.7	77	71.3
Sefwi Wiawso	44	26.7	121	73.3

^a^Timber + Non-timber species = Tree abundance, i.e. the number of stems enumerated on a total plot size of 9 ha

### Carbon stocks in cacao and shade trees in the cacao landscape

Carbon stocks in shade trees ranged from 2.91 ± 0.95 Mg C ha^−1^ at Akontombra to 15.6 ± 2.89 Mg C ha^−1^ at New Edubiase and differed significantly (F = 4.51; p = 0.0001) between districts (Table 5). Carbon stocks in cacao trees on the other hand were similar (F = 1.73; p = 0.095) and averaged 7.45 ± 0.41 Mg C ha^−1^ (range 5.84–10.2 Mg C ha^−1^). Total carbon (shade + cacao trees) significantly differed (F = 3.82; p = 0.0005) between sites with New Edubiase having the highest (23.4 ± 3.23 Mg C ha^−1^) and Sefwi Wiawso the lowest (10.9 ± 1.56 Mg C ha^−1^), representing 85.9 and 40.0 Mg CO_2_-eq. ha^−1^ respectively. Carbon distribution between cacao and shade trees averaged of 48 and 52 % respectively and mean stocks across all study districts stood at 15.5 ± 1.19 Mg C ha^−1^.

**Table 5:** Mean values ± SEM of shade tree carbon, cacao tree carbon, total carbon and their CO_2_ equivalents in ten cacao growing districts in the Ashanti, Brong-Ahafo and Western regions of Ghana (numbers in parenthesis are standard errors of the means)

District	Shade trees C (Mg C ha^−1^)	Cacao tree C (Mg C ha^−1^)	Total C (Mg C ha^−1^)	CO_2_ equiv. of total C (Mg CO_2_-eq. ha^−1^)
Sefwi Wiawso	5.05 ± 1.40^ab^	5.84 ± 0.91^a^	10.9 ± 1.56^b^	40.0
Asempanaye	11.8 ± 1.99^a^	6.77 ± 0.68^a^	18.5 ± 2.12^ab^	67.9
Akontombra	2.91 ± 0.95^b^	10.2 ± 1.31^a^	13.2 ± 2.07^ab^	48.4
Adabokrom	7.70 ± 1.29^ab^	8.02 ± 0.87^a^	15.7 ± 0.87^ab^	57.6
Sankore	8.60 ± 1.92^ab^	7.00 ± 0.87^a^	15.6 ± 2.40^ab^	57.2
Goaso	9.17 ± 2.33^ab^	7.79 ± 0.74^a^	15.4 ± 2.58^ab^	56.5
Asumura	5.04 ± 1.35^ab^	7.58 ± 0.47^a^	12.6 ± 1.68^ab^	46.2
New Edubiase	15.6 ± 2.89^a^	6.28 ± 0.82^a^	23.4 ± 3.23^a^	85.9
Offinso	9.29 ± 1.41^a^	8.65 ± 0.70^a^	17.9 ± 1.45^ab^	65.7
Nkawie	8.02 ± 0.65^ab^	6.40 ± 0.61^a^	11.5 ± 1.19^b^	42.2

Figures in the same column followed by similar alphabets do not differ significantly (p ≤ 0.05) based on the Kruskal–Wallis parametric ANOVA applied to mean ranks. Total carbon stock was converted to tons of CO_2_ equivalent by multiplying by 44/12 or 3.67 [23]

### Relationships between crown cover, diversity indices and carbon stocks

**Fig. 3:**
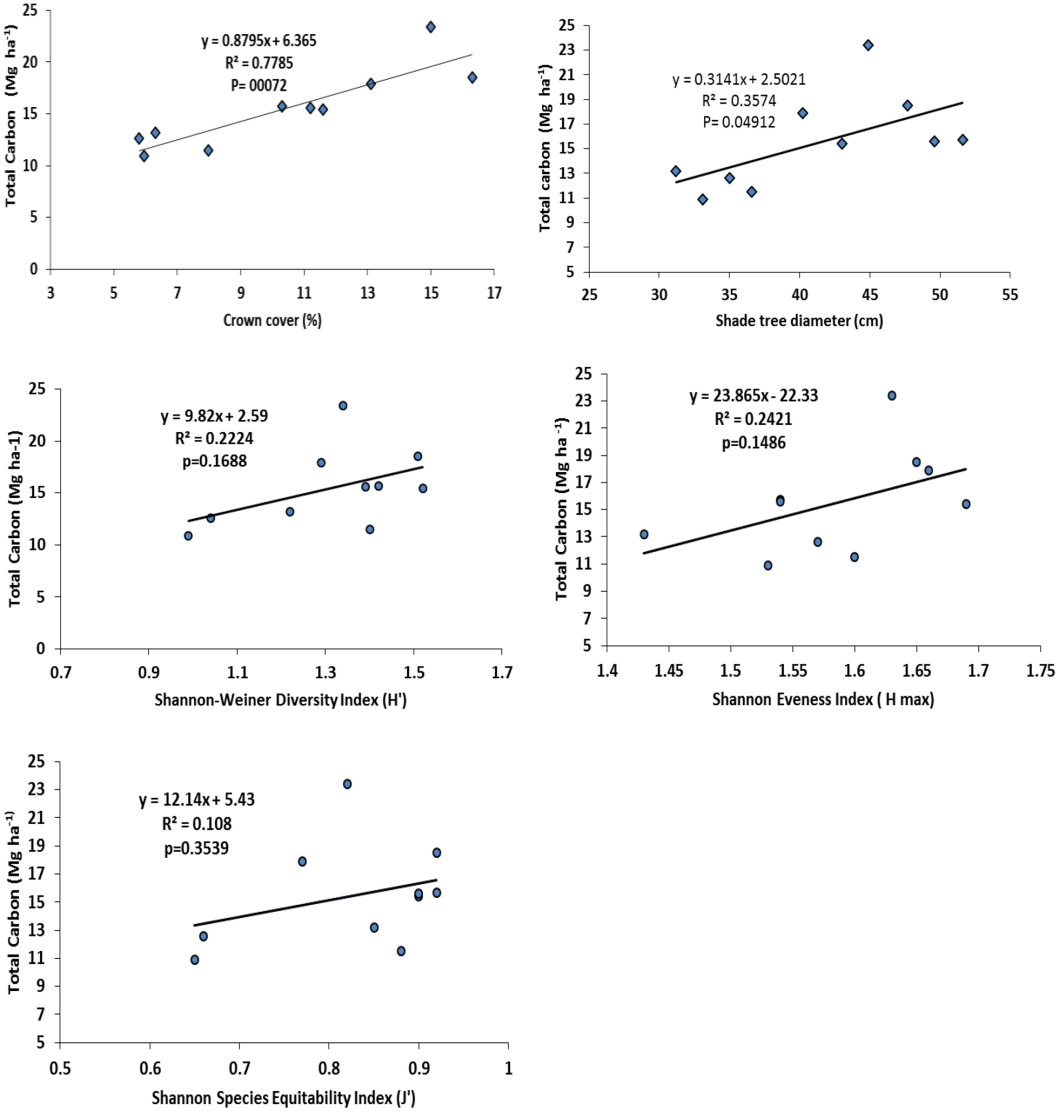
Relationships between selected shade tree dendrometric and diversity parameters and total aboveground (shade + cacao trees) carbon

We analyzed the relationships and determined correlations between various shade tree parameters, diversity indices and total aboveground tree carbon to understand how these parameters affected carbon accumulation in shaded-cacao systems. Significant linear relationships were found to exist between crown cover % and total carbon C stocks (R^2^ = 0.7785; p = 0.0072), and shade tree diameter and total carbon (R^2^ = 0.3574, p = 0.04912) (Fig. 2). The relationships between total carbon and Shannon-Weiner Diversity Indices H’, H max and J’ were not significant, p values being 0.2224, 0.1486 and 0.3539 respectively (Fig. 3). Apart from the equation relating crown cover % and total carbon stocks which had a moderately high predictive ability (R^2^ = 0.7785), all the other regressions equations explained up to a maximum of 35 % of the variations in the relationships. Crown cover (%) was highly significantly (p < 0.01) correlated to shade tree C and total C (Table 6). Whereas Shannon H max correlated significantly (p < 0.05) positively with shade tree C and number of shade trees, it was non-significantly correlated with total C. Number of shade trees correlated positively but non-significantly (p > 0.05) with total C shade tree C and Crown  %.

**Table 6:** Pearson correlation coefficients among diversity indices, number of shade trees and C content of trees in ten cacao growing districts in Ghana

	Crown %	Shannon H’	Shannon H max	Shannon J	Shade trees C	Total C
Shannon H’	0.7107*	–				
Shannon H max	0.6788*	0.4994 ns	–			
Shannon J	0.5269 ns	0.9333**	0.1556 ns	–		
Shade trees C	0.8952**	0.5791 ns	0.7078*	0.3692 ns	–	
Total C	0.8823**	0.4716 ns	0.4920 ns	0.3286 ns	0.8807**	
No. of shade trees	0.2988 ns	−0.2202 ns	0.6467*	−0.5190 ns	0.4560 ns	0.3595 ns

*ns* not significant

Asterisks denote statistical significance of correlations: * p < 0.05; ** p < 0.01

### Carbon stocks distribution and associated shade tree parameters

Shade tree characteristics (mean number of stems ha^−1^, average height (m), average stem diameter (cm) and associated total C stocks (Mg C ha^−1^) grouped according to districts with similar crown cover (%) are given in Table 7. Three groups of districts are discernible. Group 1 districts having crown cover <8 %, Group 2 districts 8.1–14.9 % and Group 3 districts have crown cover >15 %. Group 3 districts, i.e. New Edubiase and Asempanaye with the highest number of stems ha^−1^ and stem diameter recorded the highest mean carbon stocks of 21.0 ± 2.45 Mg C ha^−1^ because the biggest trees were found on these landscapes. Group 1 districts with fewer and smaller trees recorded the lowest total carbon storage of 12.1 ± 0.52 Mg C ha^−1^. Progressively also, total tree carbon increased with increasing crown cover. Group 3 districts with crown cover equal to or more than the 15 % (the defined national threshold for forests in Ghana) compared to the other sites translated to high total tree carbon stocks.

**Table 7:** Shade tree dendrometric parameters and associated carbon stocks in the study districts with similar crown cover

Group	District	Crown cover %	Mean shade trees stem number (ha^−1^)	Mean shade tree height (m)	Mean stem diameter (cm)	Shade and cacao trees C-stocks (Mg C ha^−1^)
1	Asumura	5.80	16.3	13.9	35.0	12.6
	Sefwi Wiawso	5.95	18.3	13.7	33.1	10.9
	Akontombra	6.31	9.3	11.9	31.2	13.2
	Nkawie	7.97	15.1	12.8	36.6	11.5
	Mean ± SE	6.51 ± 0.49	14.7 ± 1.93	13.1 ± 0.46	34.0 ± 1.17	12.1 ± 0.52
	Range	5.80–8.0	9.3–18.3	11.9–13.9	31.2–36.6	10.9–13.2
2	Adabokrom	10.3	10.8	15.1	51.6	15.7
	Sankore	11.2	12.0	18.2	49.6	15.6
	Goaso	11.6	16.2	15.3	43.0	15.4
	Offinso	13.1	22.8	13.5	40.2	17.9
	Mean ± SE	11.6 ± 0.58	15.4 ± 2.7	15.5 ± 0.98	46.1 ± 2.69	16.2 ± 0.587
	Range	8.1–14.9	10.8–22.8	13.5–18.2	40.2–51.6	15.4–17.9
3	New Edubiase	15.0	20.6	18.2	44.9	18.5
	Asempanaye	16.3	14.2	19.1	47.7	23.4
	Mean ± SE	15.5	17.4 ± 3.20	18.7 ± 0.45	46.3 ± 1.40	21.0 ± 2.45
	Range	≥15.0	14.2–20.6	18.2–19.1	44.9–47.7	18.5–23.5

## Discussions

### Abundance and composition of upper canopy tree species and families in the cacao landscape

Several workers (e.g., [24–26]) have emphasized that agrobiodiversity is an important feature of agro-ecological systems in terms of climate change adaptation. Shaded-cacao agroforestry may be considered promising in this context. Shade tree diversity in cacao farms offers farmers an array of agronomic, economic, cultural, and ecological benefits [9]. A diversified farm also enables farmers to exploit the different components as well as their interactions in the system to meet subsistence needs, maximize incomes, and reduce risks against fluctuations in world market prices of cacao beans [27–29]. The 109 species (DBH ≥ 15 cm) with Shannon-Weiner diversity index H’ ranging from 0.99 to 1.54 recorded from 90 ha of shaded-cacao systems in this study are relatively lower compared to values recorded in similar systems elsewhere. For instance, [30] found 293 tree species (DBH ≥10 cm) with Shannon-Weiner diversity indices ranging from 3.31 to 4.22 on 15 ha of five traditional cacao growing (*cabruca*) farms in Southern Bahia, Brazil, while in the dense and complex agroforestry systems of Southeast Cameroon. [31] sampled trees and pseudo-trees (e.g., banana) with DBH >2.5 cm and found 206 species in 60 cacao farms, with Shannon diversity indices ranging between 3.1 and 4.2 in each of the agroforestry systems studied. At two sites in the Atwima District of the Ashanti Region of Ghana, [13] recorded a Shannon index of 4.69 for matured shaded-cacao farms. [13] study area falls within the same district as the study site for this study, which recorded a significantly lower tree diversity index of 0.88. It is likely that there has been an increased removal of shade trees in the district within the intervening 3-year period prior to this study as a result illegal chainsaw operations and the continuous giving out of these off-reserve areas to timber concessionaires [18]. Our species number is however comparable to that of [32] who recorded 105 species in *cabrucas* of the Espirito Santo state, Brazil, by sampling trees with DBH >10 cm in 4.8 ha of 20 farms studied.

### Relative abundance of timber and non-timber species in the cacao landscape

Most of the sites recorded wide disparities in the abundance of timber and non-timber tree species, a strong indication that farmers are shifting the composition of species in cacao agroforestry systems in favor of non-timber species. This disparity could also be a manifestation of logging impact or exploitation of timber species within the cacao landscape. It is obvious from the data that there is a shift in the species composition of the off-reserve landscape in the high forest zone, with a general decline in species diversity. The dominance of fruit trees (citrus and avocadoes) and *Newbouldia laevis* strongly indicates a deliberate transformation of the landscape by farmers from the naturally occurring pioneer species such as *Terminalia* spp. and other timber species that have been traditionally grown in tandem with cacao, and provide timber benefits, to non-timber species such as fruits, *Gliricidia sepium* and *N. laevis*. A prime factor that drove the transformation of the species composition of the landscape was logging [18]. However, general governance issues such as tree tenure and ownership and inconsistent understanding and weak implementation of forest policy and legal regime have also contributed to a strong negative perception about tree incorporation in the cacao systems.

Farmers consistently complained about the indiscriminate logging on their farms and continued award of concessions in the cacao landscape as well as the lack of proper compensation for cacao trees destroyed [18]. For a long time, the governance issues associated with off-reserve trees have bedeviled efforts at improving cacao agroforestry systems involving the incorporation of timber trees that could have multiple benefits for farmers in Ghana. The minimal occurrence and low dominance of the indigenous timber species such as *Terminalia* spp., *Melicia excelsa*, etc. within the cacao landscape have serious implications for the implementation of climate-smart cacao models and REDD+ strategies. Most of these models are posited on indigenous timber species that present better opportunities to contribute to climate change mitigation projects in cacao systems and also serve as preferred species in terms of shade and moderation of climatic parameters for optimal cacao productivity. In order to reverse the current trend in terms of dominant species within the landscape, it is important to overcome the governance issues associated with off-reserve timber trees exploitation at all levels, from the national to the community level. Once farmers’ confidence has been gained, then mechanisms can be rolled out to introduce tree diversification strategies. It is also possible that the dominance of fruit trees is an indication that farmers want to diversify the sources of income as was indicated by respondents at Offinso, who incorporated citrus and pear to ensure that they could generate revenue in the off season period of cacao harvesting.

### Carbon stocks in cacao and shade trees

Agroforestry ecosystems are generally known to stock higher carbon than other cropping systems thereby contributing to climate change mitigation [33–35]. Closed forests in the study area are generally reported to hold about 155 Mg C ha^−1^ with stocks in open forests ranging in the region between 85 and 96 Mg C ha^−1^, while agricultural lands including no-shade cocoa systems stock on the average 15 Mg C ha^−1^ [14, 36, 37]. Thus, the conversion of agricultural lands to cacao agroforests could be a management strategy for storing large quantities of carbon. As the largest component of total biomass, shade trees are crucial to carbon stocks in the cacao cultivation systems. Our findings of higher stocks in some districts compared to others reflects emerging or existing trends in the management of upper canopy trees and how this holds implications for landscape level carbon build-up or loss.

Our results for total tree carbon stocks in cocoa agroforests are similar to and fall within the range reported by [14] in the high forest cacao zone in the Western Region in Ghana, which is also similar to that of [38] in Central Sulawesi and [39] in Central Cameroon. [14] recorded aboveground carbon stocks of 16.8 and 15.9 Mg C ha^−1^ respectively in 15 and 25-year-old shaded-cacao stands. Results of this study however fall in the lower range compared to studies by [40] in Bolivia, [7, 11, 39, 41] all in Cameroon, [36] in Ghana and [42] in Sulawesi, Indonesia. In these studies, stocks of tree carbon ranged from 34.4 to 135.5 Mg C ha^−1^. Explanations for the lower carbon stocks found in this study include the relatively young age of most of the plots (40.7 % of all farms were 10 years or below) as well as the non-planting and removal of shade trees due to the perception that, shade trees are a major cause of pests, diseases and low yields. It also needs to be pointed out that, aboveground carbon depends on a number of factors including the canopy species, tree density, environment and the approach used in estimating total carbon. [7] have pointed out that using equations like [43] which do not incorporate wood density would overestimate the biomass and thus carbon stocks of a low wood density species such as cacao. Using allometric equations that integrate wood densities of separate species, as done in the current study, or developing location and species-specific allometric equations is likely to lead to a more accurate assessment of biomass and thus carbon stocks. This study used the equation developed by [19]. Hence, comparison of the carbon stocks figures generated in this study should also be placed in the context of the allometric equations used in the estimation of biomass. But most obviously, the fact that this study adopted a transect approach, also means that, all variations of cacao maturity stages and establishment were encountered. Given that biomass build-up directly relates to age, further comparisons of our results, should be placed within the context of random transect sampling.

### Relationships between shade tree dendrometric parameters and carbon stocks

Associated shade tree parameters (crown cover and shade tree stem diameter) significantly influenced aboveground C stocks in the cacao agroforestry systems. The predictive ability of the equation relating crown cover and c-stocks is moderately high while that between shade tree stem diameter and shade tree carbon and total tree carbon were generally low, the equations nevertheless demonstrate the relationship between the size of the shade tree and the amount of carbon stored. Progressively, total tree carbon increased with increasing crown cover. This is to be expected as increasing tree size (tree height and diameter) is generally associated with increasing crown cover. The bigger the stem diameter, the more the amount of carbon stored. It has been widely recognized that, shade trees in cacao systems account for the differences in carbon stocks in cacao systems. Though they are often assumed to negatively affect growth and yield of cacao plants through competitive water [44] and resource use, shade trees play a significant role on C sequestration. Empirical studies have also shown the positive effects of plant species-specific, complementary resource use in agroforestry systems [45]. Several authors (e.g. [6, 44]) have emphasized that the main drivers of C storage in cacao systems are shade trees, which usually has the highest amount of carbon. Studies by [6, 7] for instance all recorded shade trees storing up to 65 % of total tree carbon in cacao systems studied in Nigeria and Cameroon respectively.

The near-even distribution of carbon between cacao (48 %) and shade trees (52 %) in this study is possibly a reflection of the extent to which shade trees have been and continue to be progressively removed from farms. This could be the result of the widely held perception by farmers in some districts that presence of shade trees on farms is a major cause of disease incidence and low yields. There are also governance issues particularly that which relates tenure over naturally growing economic trees in cacao and other cash crop plantations. Existing tree tenure system where farmers have no ownership right over naturally growing economic trees in their cacao farms, has served as a disincentive for farmers to nurture and keep economic trees in cacao farms. When combined with a preference for full-sun cacao over shade production systems, as a result of relatively high yields obtained in the initial years of cultivation [10], there appears to be a strong incentive to remove shade trees from cacao farms in these and several other cacao growing districts [18]. However, since the government introduced the timber resources management (Amendment) Act of 2002, the right of tenure has now been assigned to the one who planted the tree, though this law appears to be more inclined towards plantation establishment and has largely not been tested in farming systems. This provides hope for REDD+ implementation in cacao farms, as this could motivate farmers to integrate trees into their farms through planting, if appropriate sensitization efforts are made, including enforcement of existing laws that that require that farmers consent is sought before trees are logged.

### Possibility of implementing REDD+ interventions in studied cacao landscapes

Results of this study also reveal that, contrary to existing assumptions, unshaded cacao systems meet the height requirements to qualify as forest per Ghana’s forest definition for climate change mitigation projects. Coupled with the finding that only two of the studied districts have shade cover equal to or more than the 15 % threshold, significant opportunity exists to develop cacao-carbon and REDD+ projects in Ghana. This is certainly useful information and has diverse implications for the design of Ghana’s REDD+ strategy. However, the extent to which cacao landscapes would contribute to the realization of REDD+ objectives hinges very much on Ghana’s definition of forest. In line with requirements under the REDD+ readiness efforts, Ghana defined its forests as being a minimum of 1 ha, having at least 15 % canopy cover and containing trees that are 5 meters tall. The shade trees in the cacao system, however, could constitute a forest if they offer enough canopy cover beyond the 15 % and are taller than 5 m. Thus, the forest definition and type of cacao system (monoculture vs. shade) have serious implications for the type of REDD+ that is viable. Two out of the ten study districts, New Edubiase and Asempaneye, had crown cover of 15 and 16.3 % respectively and technically qualify to be defined as forests and inclusion for REDD+ intervention activities. By judiciously selecting shade trees and integrating them into cacao systems in a manner designed to offer optimal ecological, economic nutritional and financial benefits, cacao landscapes in the other study districts have the potential of contributing positively to the country’s REDD+ efforts. Full-sun plantations with cacao trees up to the defined 5-m height would constitute a forest and therefore would qualify for REDD+ .

## Conclusions and recommendations

There were significant relationships between crown cover (%) and shade tree carbon stocks, shade tree stem diameter and shade tree carbon, and shade tree diameter (cm) and total aboveground carbon stocks. Progressively also, total tree carbon stocks increased with increasing mean stem numbers per hectare and stem diameter. Above ground carbon stocks in shaded-cacao systems in the studied landscape varied between 10.9 ± 1.56 and 23.4 ± 3.23 Mg C ha^−1^ and is a reflection of the differences in tree density, tree diversity and management practices between farms. Additionally, only 20 % of cacao in the sampled districts is grown under shade regimes of 15 % crown cover and above, while 80 % of cacao is grown under shade regimes ranging from 5.8 to 14.9 %. In the light of these findings, significant opportunity exists to develop cacao carbon projects in Ghana. While different groups are making efforts to map out farms in the cacao landscape, comprehensive data and information such as those suggested above is needed to develop a well-planned national REDD+ strategy. By judiciously selecting preferred shade trees and integrating them into cacao systems in a manner designed to offer optimal ecological and socio-economic benefits, cacao landscapes in Ghana have the potential of contributing positively to the country’s REDD+ efforts. There is also the need to educate farmers on the potential of monetary benefits from REDD+ activities. Broad stakeholder participation and access to credit and information are essential for the equitable and sustainable implementation of REDD+ policies. Increased participation is particularly important in terms of maximizing local knowledge and capacity to support the implementation of REDD+. However, the extent to which cacao landscapes would contribute to the realization of REDD+ objectives hinges very much on Ghana’s definition of forest.

## Additional file

**Additional file 1: Appendix 1.** Shade tree species found in *Theobroma cacao* agroforestry systems in ten districts in the Ashanti, Brong-Ahafo and Western Regions of Ghana and reasons for their preference. (Uses: 1. Timber, 2. Food & beverages, 3. Fodder, 4. Fuelwood/Charcoal, 5. Soil fertility/Green manure, 6. Nitrogen-fixation, 7. Dyes/Colors, 8. Spices, 9. Honey production/Apiculture 10. Essential Oils, 11. Medicinal, and 12. Other uses).
