# Supplementary material for: Shade tree diversity and aboveground carbon stocks in Theobroma cacao agroforestry systems: implications for REDD+ implementation in a West African cacao landscape
Source: Carbon Balance Manag. 2016 Aug 24;11:17. doi: 10.1186/s13021-016-0061-x (PMC4996877; doi:10.1186/s13021-016-0061-x)
Supplement: Supplementary file 1 — Additional file 1: Appendix 1. Shade tree species found in Theobroma cacao agroforestry systems in ten districts in the Ashanti, Brong-Ahafo and Western Regions of Ghana and reasons for their preference. (Uses: 1. Timber, 2. Food & beverages, 3. Fodder, 4. Fuelwood/Charcoal, 5. Soil fertility/Green manure, 6. Nitrogen-fixation, 7. Dyes/Colors, 8. Spices, 9. Honey production/Apiculture 10. Essential Oils, 11. Medicinal, and 12. Other uses). [file 13021_2016_61_MOESM1_ESM.docx]

**APPENDIX 1**

Shade tree species found in *Theobroma cacao* agroforestry systems in ten districts in the Ashanti, Brong-Ahafo and Western Regions of Ghana and reasons for their preference. (Uses: 1. Timber, 2. Food & beverages, 3. Fodder, 4. Fuelwood/Charcoal, 5. Soil fertility/Green manure, 6. Nitrogen-fixation, 7. Dyes/Colors, 8. Spices, 9. Honey production/Apiculture 10. Essential Oils, 11. Medicinal, and 12. Other uses).

| **Botanical/Scientific Name** | **Family** | **Uses** |
| --- | --- | --- |
| *Afzelia bella* | Caesalpiniaceae | 1, 3, 4, 11, |
| *Albizia adianthifolia* | Mimosaceae | 4, 3, 11 |
| *Albizia ferruginea* | Mimosaceae | 1, 4, |
| *Albizia glaberrima* | Mimosaceae | 3, 4, |
| *Albizia zygia* | Mimosaceae | 1, 4 |
| *Alstonia boonei* | Apocynaceae | 1, 4, 11 |
| *Amphimas pterocarpoides* | Papilionaceae | 1, 4, 11 |
| *Anacardium occidentale* | Anacardiceae | 1, 2, 4, 7, 10,11 |
| *Antiaris toxicaria* | Moraceae | 1, 2, 3, 4 |
| *Antrocaryon micraster* | Anacardiaceae | 1, 11, 4 |
| *Aubrevillea kerstingii* | Mimosaceae | 1, 4, 11 |
| *Baphia pubescens* | Papilionaceae | 4, 7, 11 |
| *Beilschmiedia mannii* | Lauraceae | 4, 11, 12 |
| *Blighia sapida* | Sapindaceae | 2, 4, 11, 12 |
| *Blighia unijugata* | Sapindaceae | 2, 4, 11 |
| *Bombax buonopozense* | Bombacaceae | 1, 3, 4, 11, 12 |
| *Calpocalyx brevibracteatus* | Mimosaceae | 4, 11, 12 |
| *Canarium schweinfurthii* | Burseraceae | 1, 4, 2, 10, 12 |
| *Carapa procera* | Meliaceae | 11, 4, 12 |
| *Cecropia peltata* | Cecropiaceae | 11 |
| *Cedrela odorata* | Meliaceae | 1, 4, 9, 11 |
| *Ceiba pentandra* | Bombacaceae | 1,11, 12 |
| *Celtis mildbraedii* | Ulmaceae | 1, 4, 11 |
| *Celtis zenkeri* | Ulmaceae | 1, 4, 11 |
| *Chrysophyllum perpulchrum* | Sapotaceae | 4, 11 |
| *Chrysophyllum ssp* | Sapotaceae | 4 |
| *Chrysophyllum subnidum* | Sapotaceae | 2, 11, 4 |
| *Citrus reticulata* | Rutaceae | 2, 4, 10, 11 |
| *Citrus sinensis* | Rutaceae | 2, 4, 10, 11 |
| *Cocus nucifera* | Arecaceae | 2, 10, 11, 12, |
| *Cola caricifolia* | Sterculiaceae | 4, 11 |
| *Cola gigantea* | Sterculiaceae | 1, 4, 11 |
| *Cola nitida* | Sterculiaceae | 2, 4, 7, 11, 12 |
| *Cordia millenii* | Boraginaceae | 4, 11, 12 |
| *Daniella ogea* | Caesalpiniaceae | 1, 4, 11 |
| *Dialium dinklagei* | Caesalpiniaceae | 4, |
| *Dialium guineensis* | Caesalpiniaceae | 2, 4 |
| *Discoglypremna caloneura* | Euphorbiaceae | 4 |
| *Distermonanthus benthamianus* | Caesalpiniaceae | 1, 4, 13 |
| *Dracaena mannii* | Dracaenaceae | 7, 11,12 |
| *Elaeis guineensis* | Arecaceae | 2, 10, 12 |
| *Entandrophragma angolense* | Meliaceae | 1, 4, 11, |
| *Entandrophragma candollei* | Meliaceae | 1, 4 |
| *Erythrina vogelii* | Papilionaceae | 11, 4 |
| *Ficus carpensis* | Moraceae | 4 |
| *Ficus exasperata* | Moraceae | 3, 4 |
| *Ficus sur* | Moraceae | 4, 11 |
| *Ficus trichopoda* | Moraceae | 4 |
| *Ficus vogelii* | Moraceae | 4 |
| *Funtumia elastica* | Apocynaceae | 4, 11, 12 |
| *Gliricidia sepium* | Papilionaceae | 3, 6 |
| *Gmelina arborea* | Verbenaceae | 3, 12 |
| *Guibortia leonensis* | Caesalpiniaceae | 4 |
| *Hannoa klaineana* | Simaroubaceae | 1, 4 |
| *Harungana madagascariense* | Guttiferae | 4 |
| *Holarrhaena floribunda* | Apocynaceae | 1, 4, 12 |
| *Khaya anthotheca* | Meliaceae | 1, 3, 4, 11 |
| *Khaya ivorensis* | Meliaceae | 1, 3, 4, 11 |
| *Lannea welwitschii* | Anacardiaceae | 1, 4, 12 |
| *Lecaniodiscus cupanoides* | Sapindaceae | 4 |
| *Lonchocarpus sericeus* | Papilionaceae | 4 |
| *Macaranga barteri* | Euphorbiaceae | 4 |
| *Macaranga heterophyla* | Euphorbiaceae | 4 |
| *Mangifera indica* | Anacardiaceae | 2, 3, 4, 11 |
| *Mansonia altissima* | Sterculiaceae | 1, 4 |
| *Margaritaria discoidea* | Euphorbiaceae | 4 |
| *Milicia excelsa* | Moraceae | 1, 4 |
| *Millettia rhodentha* | Papilionaceae | 4 |
| *Monodora myristica* | Annonaceae | 4, 8, 10, 11 |
| *Morinda lucida* | Rubiaceae | 4, 7, 11 |
| *Morus mesozygia* | Moraceae | 1, 4, 12 |
| *Myrianthus arboreus* | Cecropiaceae | 2, 4, 11 |
| *Nauclea diderrichii* | Rubiaceae | 1, 4 |
| *Nauclea latifolia* | Rubiaceae | 4 |
| *Nesogordonia papaverifera* | Sterculiaceae | 1, 4 |
| *Newbouldia laevis* | Bignoniaceae | 11 |
| *Okuobaka aubrevillei* | Santalaceae | 2, 4 |
| *Persea americana* | Lauraceae | 2 |
| *Petersianthus marcrocarpus* | Lecythidaceae | 1, 4 |
| *Pierreodendron kerstingii* | Simaroubaceae | 4 |
| *Piptadeniastrum africanum* | Mimosaceae | 1, 4 |
| *Pouteria altissima* | Sapotaceae | 1, 4 |
| *Psidium guajava* | Myrtaceae | 2, 4 |
| *Pterocarpus santalinoides* | Papilionaceae | 4 |
| *Pterygota macrocarpa* | Sterculiaceae | 1 |
| *Pycnanthus angolensis* | Myristicaceae | 1 |
| *Rauvolfia vomitoria* | Apocynaceae | 4 |
| *Rhodognaphalon brevicuspe* | Bombacaceae | 1 |
| *Ricinodendron heudelotii* | Euphorbiaceae | 4, 11 |
| *Scottellia klaineana* | Achariaceae | 4 |
| *Spathodea campanulata* | Bignoniaceae | 4, 11 |
| *Spondias mombin* | Anacardiaceae | 2, 4 |
| *Sterculia oblonga* | Sterculiaceae | 1, 11 |
| *Sterculia rhinopetala* | Sterculiaceae | 1 |
| *Sterculia tragacantha* | Sterculiaceae | 11 |
| *Strombosia glaucescens* | Olacaceae | 1, 11 |
| *Terminalia ivorensis* | Combretaceae | 1 |
| *Terminalia superba* | Combretaceae | 1 |
| *Tetrapleura tetraptera* | Mimosaceae | 2 |
| *Treculia africana* | Moraceae | 2, 11 |
| *Trema orientalis* | Ulmaceae | 2, 3, 7, 11 |
| *Trichilia monadelpha* | Meliaceae | 11 |
| *Trilepisium madagascariense* | Moraceae | 11 |
| *Triplochiton scleroxylon* | Sterculiaceae | 1 |
| *Turraeanthus africanum* | Meliaceae | 1 |
| *Vernonia amygdalina* | Asteraceae | 11 |
| *Vitex ferruginea* | Verbenaceae | 11 |
| *Voacanga africana* | Apocynaceae | 11 |
| *Zanthoxyllum gillettii* | Rutaceae | 1, 11, 12 |
